# Effects of general anesthetics on visceral pain transmission in the spinal cord

**DOI:** 10.1186/1744-8069-4-50

**Published:** 2008-10-30

**Authors:** Yun Wang, Jing Wu, Qing Lin, HJ Nauta, Yun Yue, Li Fang

**Affiliations:** 1 Department of Anesthesiology, Beijing Chaoyang Hospital, Capital Medical University, Beijing 100020, PR China; 2 Department of Neuro-Oncology, the University of Texas MD Anderson Cancer Center, Houston, TX 77030, USA; 3 Division of Neurosurgery, Department of Surgery, the University of Texas Medical Branch, Galveston, TX 77555-0517, USA; 4 Department of Neuroscience and Cell Biology, the University of Texas Medical Branch, Galveston, TX 77555-0517, USA

## Abstract

Current evidence suggests an analgesic role for the spinal cord action of general anesthetics; however, the cellular population and intracellular mechanisms underlying anti-visceral pain by general anesthetics still remain unclear. It is known that visceral nociceptive signals are transmited via post-synaptic dorsal column (PSDC) and spinothalamic tract (STT) neuronal pathways and that the PSDC pathway plays a major role in visceral nociception. Animal studies report that persistent changes including nociception-associated molecular expression (e.g. neurokinin-1 (NK-1) receptors) and activation of signal transduction cascades (such as the protein kinase A [PKA]-c-AMP-responsive element binding [CREB] cascade)-in spinal PSDC neurons are observed following visceral pain stimulation. The clinical practice of interruption of the spinal PSDC pathway in patients with cancer pain further supports a role of this group of neurons in the development and maintenance of visceral pain. We propose the hypothesis that general anesthetics might affect critical molecular targets such as NK-1 and glutamate receptors, as well as intracellular signaling by CaM kinase II, protein kinase C (PKC), PKA, and MAP kinase cascades in PSDC neurons, which contribute to the neurotransmission of visceral pain signaling. This would help elucidate the mechanism of antivisceral nociception by general anesthetics at the cellular and molecular levels and aid in development of novel therapeutic strategies to improve clinical management of visceral pain.

## Introduction

Visceral pain is the most common sign of acute and chronic gastrointestinal, pelvic, genitourinary, and other internal solid-organ diseases. When visceral structures are stretched, compressed, inflamed, or distended, a poorly localized noxious visceral feeling is reported. As one of the most common causes of long-term suffering and persistent disability, this represents a frequent reason for patients to seek medical treatment. Despite multiple therapeutic approaches, the medical community still faces a significant challenge to relieve acute and chronic visceral pain effectively, especially in cancer patients with pain. On the other hand, as practical anesthesiology extends itself into peri-operative pain treatment, the anesthesiologist's expertise in the management of intra-operative visceral pain and intractable or cancer-related visceral pain is highly valued [1]. For example, many diagnostic and therapeutic procedures, such as gastrointestinal and genitourinary endoscopies are associated with visceral organs, which can cause acute visceral nociception and may require general anesthetic administration including infusion of propofol or inhalation of sevoflurane. However, little is known regarding the spinal mechanisms underlying the inhibition of visceral nociception by general anesthetics.

It has been demonstrated that the spinal cord is one of the critical working targets of general anesthetics [2,3]. A study indicates that general anesthetics, such as propofol and isoflurane, may affect different cellular populations in the spinal cord to produce analgesia and immobility [4]. Several ascending tracts originating from the spinal cord such as the spinothalamic, spinohypothalamic, spinoreticular, spinoparabrachial, spinomesencephalic, spinosolitary, and spinolimbic tracts have been shown to play roles in transmission of noxious somatic and visceral information [5]. Additionally, recent investigations from bench and bedside by our group suggest that a critical visceral nociceptive pathway originates from PSDC neurons located in the central area of the spinal cord [6-8]. Interruption of the PSDC pathway using different surgical approaches relieves intractable visceral pain in cancer patients [9-15]. Therefore, based on current laboratory and clinical findings, we hypothesize that general anesthetics exert an inhibitory effect on visceral nociception via the PSDC pathway. Investigation of inhibition of the PSDC pathway by general anesthetics will identify a neurobiological mechanism of general anesthetic action and should help in the development of novel therapeutic strategies for visceral pain management. This review will summarize the effects of general anesthetics in blocking visceral pain with a focus on the role of the spinal PSDC pathway.

### Role of the PSDC pathway and PSDC neurons in the transmission of visceral nociception

Traditionally, the STT is believed to be the most important nociceptive pathway, while the dorsal column (DC) system is usually considered to be involved in signaling information concerning innocuous stimuli [16]. However, several clinical and experimental studies have provided compelling evidence that the DC pathway plays a critical role in relaying visceral nociceptive information [6-8,17-19]. In clinical settings, transection of the lateral column of the spinal cord does not provide effective visceral pain relief, while the interruption of DC leads to considerable relief of intractable visceral pain in cancer patients [6,7]. Electrophysiological experiments in laboratory animals showed that a lesion of the DC or DC nuclei in medullar oblongata significantly diminished the increased activity of thalamic ventroposteriolateral nuclei evoked by noxious visceral stimuli [20,21]. Behavioral studies in mice demonstrated that a high cervical midline punctate myelotomy apparently decreased the somatic responses to the intraperitoneal injection of acetic acid [22]. The reduction of exploratory activity present after the capsaicin injection could be prevented by ipsilateral dorsal rhizotomy or a contralateral lesion of the lateral funiculus, but was not affected by a DC lesion. In contrast, a bilateral DC lesion made prior to noxious colon stimulation counteracted the decrease in exploratory activity observed in naïve animals, and this effect could last up to 180 days following the interruption of the DC pathway [23]. These findings confirmed an important role of the PSDC pathway in visceral nociceptive neurotransmission.

**Figure 1:**
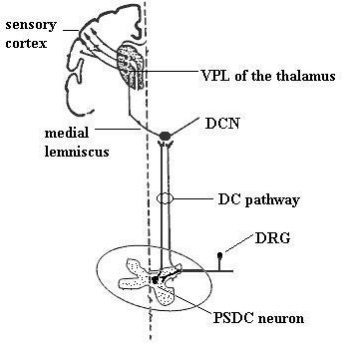
**Dorsal column pathway of visceral pain transmission.** Adapted from Nauta HJ, et al. Surgical interruption of a midline dorsal column visceral pain pathway. J Neurosurg, 86:538–542, 1997. The dorsal column pathway is composed of branches of primary afferent fibers, some of which project directly to the dorsal column nuclei, and of the axons of postsynaptic dorsal column neurons. Pelvic viscera nociceptive input activates the postsynaptic dorsal column neurons of the spinal cord and is relayed to higher centers. PSDC neurons receiving pelvic visceral input send their axons in the midline of the dorsal column to synapse in the nucleus gracilis. Then, the pathway crosses the midline in the lower brainstem to ascend to the ventral posterolateral nucleus of the thalamus. PSDC (postsynaptic dorsal column); VPL (ventral posterolateral), DRG (dorsal root ganglion).

The DC pathway is composed of input of branches of primary afferent fibers, some of which project directly to the DC nuclei, and input of axons of PSDC neurons (Figure 1). PSDC neurons are located in the nucleus proprius and in the vicinity of the central canal in the spinal gray matter and project to the gracile and cuneate DC nuclei [24]. The activation of PSDC neurons by noxious visceral stimuli in rats was demonstrated in recent experiments [25-28]. Following noxious ureter stimulation, a higher percentage of retrogradely labeled PSDC neurons showed the expression of *c-fos *protein than of STT neurons, while no significant difference in *c-fos *expression in these two populations of neurons was detected after intradermal capsaicin injection [26]. Intraspinal application of morphine or the AMPA receptor antagonist, CNQX, to block neurotransmission at the spinal cord level prevents the activation of neurons in the gracile nucleus induced by noxious colonic distention [27]. This suggested a great contribution of PSDC neurons than of STT neurons in transmitting noxious visceral stimuli.

### Activation of signal transduction pathways in PSDC neurons in response to visceral stimuli

Noxious visceral stimulation, such as intracolonic injection of mustard oil or capsaicin, produced an enhanced responsiveness of spinal nociceptive neurons involved in triggering the activation of a number of neurotransmission molecules. An increased expression of neurokinin NK1 receptors in PSDC neurons was demonstrated after colonic inflammation or bladder irritation [25,29]. NK1 receptor expression is upregulated in the dorsal horn and in PSDC neurons after visceral stimuli [29]. Intrathecally applied NK1 antagonists significantly reduce abdominal muscle contractions induced by colon inflammation [30]. Visceral primary afferents are known to be rich in neuropeptides, such as substance P (SP), and a significant deficit in visceral nociceptive perception was observed in studies of SP-receptor (NK1) knockout mice [31,32]. These results indicate that NK1 receptors in PSDC neurons play a critical role in the transmission of visceral nociceptive signals at the spinal-cord level. Intraspinal application of an AMPA receptor antagonist, CNQX, could block the response of PSDC neurons to colonic distension, indicating that AMPA receptor activation was involved in nociceptive transmission by PSDC neurons [27].

**Figure 2:**
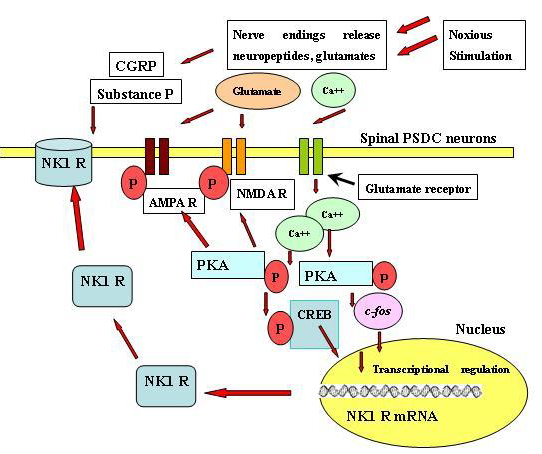
**Neurochemical signal transduction pathways in the PSDC neurons in response to visceral stimuli.** The activation of nociceptive receptors causes a large influx of calcium into the nociceptive neurons and the increased calcium influx, in turn, activates multiple intracellular protein kinases. PKA regulates the phosphorylation of glutamate receptors. Another important role for the activation of PKA in PSDC neurons is its effect on painful stimulation-elicited gene expression through mediation of transcription factors, such as *c-fos *and CREB. PKA in PSDC neurons might increase the expression of NK1 receptors through mediation of CREB and contribute to the sensitization of PSDC neurons.

It has been reported that the second messenger system conveys extracellular nociceptive signals from the plasma membrane into the nucleus of neurons in animal models of pain [33-35]. The activation of nociceptive receptors causes a large influx of extracellular calcium into nociceptive neurons; the increased calcium influx, in turn, activates multiple intracellular protein kinase cascades, such as CaM kinase II, PKA, and PKC [36-40]. Our group demonstrated the intracellular cascade in PSDC neurons mediated by PKA in a visceral pain model in rats with the intracolonic injection of mustard oil [41,42]. We found that intracolonic injection of mustard oil significantly induced the expression of PKA kinase protein, as well as CREB, in the lumbosacral spinal cord and in pre-labeled PSDC neurons. An intrathecal infusion of the PKA inhibitor, H 89, significantly blocked the visceral stimulation-induced phosphorylation of CREB protein in the spinal cord. This suggests that the PKA-mediated signal transduction cascade may contribute to visceral nociceptive transmission in PSDC neurons. It has been revealed that phosphorylation of glutamate receptors is regulated by PKA at serine/theonine residues, which are involved in central sensitization [36,37,43]. NR1 subunits of NMDA receptor and GluR1 subunits of AMPA receptors are phosphorylated by PKA. An AMPA receptor antagonist, CNQX, could block the response of PSDC neurons to colonic distension in rats [8]. To combine the finding of the increased expression of PKA in PSDC neurons, we could suggest that a possible role for PKA in regulation of AMPA receptor activity in PSDC neurons during visceral painful states. Additionally, it has been demonstrated that sex steroid hormones, such as estrogen, increased the activity of spinal NMDA receptors via PKA-mediated phosphorylation of NR1 subunits in an animal visceral pain model [44]. Another important role for the activation of PKA in PSDC neurons is that PKA may mediate painful stimulation-elicited gene expression through its regulation of transcription factors, such as *c-fos *and CREB [33,35,40,45]. Increased phosphorylation of CREB through the activated PKA was involved in CGRP-induced NK1 receptor gene expression in spinal neurons [46]. It also suggests that activation of PKA in PSDC neurons might increase CREB-induced the expression of NK1 receptor and contribute to the sensitization of PSDC neurons (Figure 2).

Another important component of secondary messenger system, PKC is widely reported to play a role in long term potentiation of spinal nociceptive neurons. Recently, our group demonstrated the phosphorylation of γ-PKC kinase in signaling visceral nociceptive transmission in PSDC neurons [47]. Interestingly, another group investigated the expression of γ-PKC isoform in spinal cord and gracile nucleus and they found that approximately 90% of γ-PKC-positive neurons in gracile nucleus and 60% in dorsal horn co-expressed GluR2/3 subunits of AMPA receptor [48]. It may link to a role for phosphorylated PKC kinases in AMPA receptor regulation in PSDC system during visceral pain states.

A family of multi-functional kinases, the mitogen-activated protein kinases (MAPKs) is critical intracellular signal mediators to regulate neuronal development and differentiation, as well as responses to extracellular stress and inflammation [49-51]. The activation of p38 MAPK was reported to contribute to the inflammatory pain and neuropathic pain [52,53]. Spinal extracellular signaling-regulated kinase-1 and -2 (ERK1/2) activation was reported to play a specific role in maintaining prolonged referred visceral hyperalgesia in adult mice [54]. Peripheral injection of hUcn 2, a corticotropin releasing factor 2 (CRF2) receptor agonist, blunts colorectal distension (CRD) induced visceral pain in an ERK 1/2 activity-dependent manner in spinal neurons [55]. However, the role of ERK activity involved in signaling visceral nociception in PSDC neurons is still unclear. Recently, phosphorylation of ERK was reported to occur in NK1 receptor-expressing neurons in laminae III-IV of rats following noxious stimulation [56]. Additionally, other critical neurotransmitters, such as NMDA and non NMDA glutamate receptors, NK1 receptors and brain-derived neurotrophic factor (BDNF) receptor were reported to be coupled to phosphorylated ERK in the dorsal horn neurons [57-59]. Combining these studies, we suggest that phosphorylation of MAP kinases might play an important role in signaling visceral information in PSDC neurons and its mechanism may be involved in the regulation of activity of glutamate and NK1 receptors in PSDC neurons [60].

These studies help to better understand the molecular signal transduction pathways in PSDC neurons under visceral pain conditions (Figure 2). Other neuronal signaling transduction pathways relaying the information involved in PSDC neurons following visceral nociceptive stimulation may deserve more investigation. These signaling transduction pathways which relay the visceral signals in PSDC neurons may serve as a development of drug target for clinical visceral pain treatment and open the possibility of replacing surgical neuroablative approaches by pharmacological agents.

### Antinociceptive effect of general anesthetics on spinal neurons

Most volatile anesthetics can induce unconsciousness, suppress autonomic responsiveness and block motor responses to noxious stimulation. Several studies have revealed that the minimum alveolar concentration (MAC) of volatile anesthetics, which is required to prevent the spontaneous mobility to nociceptive stimuli in 50% of subjects, is critically dependent on spinal-cord regulation [61-63]. The depression of motor response to noxious stimulation may be caused by immobilization and antinociceptive effects at the spinal-cord level [64]. An antinociceptive effect of halothane was observed since decreased extracellular activity of single wide-dynamic-range dorsal horn neurons was recorded in animals subjected to noxious-stimuli after inhalation of halothane [65]. Furthermore, the inhibitory effect of halothane was blocked by bicuculline, a γ-aminobutyric acid type A receptor antagonist [65]. This indicates that the antinociceptive effect of volatile anesthetics may involve γ-aminobutyric acid-mediated (GABAergic) transmission in the spinal dorsal horn. Wakai et al. also reported an antinociceptive effect of isoflurane in lamina II (substantia gelatinosa) of the spinal cord dorsal horn, which is considered to be an important structure for pain transmission [66]. They reported that isoflurane application significantly augments GABA-mediated inhibitory effects since it further reduces excitability of dorsal horn neurons [66]. Cuellar et al. compared the effects of halothane and isoflurane on lumbar dorsal horn neuronal windup and excitability [67]. They found that windup was significantly greater following isoflurane administration than after halothane anesthesia at the level of 1.2 MAC since the initial noccieptive C-fiber-mediated response was suppressed much more by using isoflurane. Volatile anesthetics may also affect the sensory functions mediated by spinal N-methyl-D-aspartate (NMDA)- and NK-1- receptors. A study showed that windup is gradually increased in spinal neurons in response to repeated stimulation of C-fibers. The data also revealed temporal summation of NMDA- and NK-1- receptor-mediated slow cumulative depolarizations of spinal neurons evoked by primary nociceptive afferent input. Other studies suggested that sevoflurane, another inhalation anesthetic, produced robust inhibitory effects on spinal GABAergic neurons in an *in vitro *experiment using isolated spinal cords [68]. Sevoflurane also modulates potassium channel conductance and depresses sensory neuronal responses mediated by glutamate receptors following noxious stimuli [69,70]. Although the spinal cord is believed to be the predominant target site where volatile anesthetics produce immobility during anesthetic procedures, the precisely targeted cellular population and specific neurobiological mechanisms remain less investigated in the setting of visceral pain states.

As one of the commonly used intravenous anesthetics in current clinical practice, propofol is widely applied in visceral procedures due to its analgesic action. An i*n vitro *experiment showed that propofol potentiated a depressant effect of opioid at a low concentration (about 0.15 μM) [71]. At clinically relevant concentration ranges (from 0.5 to 1 μM), it can produce a modest reduction of dorsal root-ventral root reflexes [72]. Matute et al. reported, in an *in vitro *preparation, that propofol had an inhibitory effect on spinal nociceptive transmission when giving at anesthetic concentrations in a hemisected rat spinal cord [68]. Using a multimodal electrophysiological assessment, Kammer et al. reported that both propofol and sevoflurane targeted preferentially the spinal cord at subanesthetic levels of dosage [73]. Nishiyama et al. reported that intrathecal propofol has analgesic effects on inflammation-induced nociception without sedative effect in rats [74]. Another *in vivo *experiment indicated that propofol had analgesic effects by depressing spinal sensitization [75]. Several clinical reports also found that propofol could reduce both H-reflexes and F-waves at subanesthetic concentrations, indicating that spinal effects of propofol could be observed [73,76]. Based on these studies from laboratories and clinics, the spinal antinociceptive effects of propofol can be confirmed.

It has been reported that several amino acid receptors are involved in the spinal antinociceptive action of propofol. Propofol works as a modulator of both GABA_A _and glycine receptors in the brain and spinal cord; these receptors play crucial roles in spinal antinociception [77,78]. Facilitation of GABA_A _and glycine receptors by propofol at the spinal-cord level might contribute to analgesia based on several studies [79-81]. Shimizu et al. reported that propofol enhanced GABA_A _receptor-mediated presynaptic inhibition at primary afferent terminals in the human spinal cord [80]. Nadeson et al. reported that intrathecal injection of a GABA_A _antagonist, dicentrine, inhibited the analgesic action of propofol in a dose-dependent manner [81]. These studies reveal a GABA_A _receptor-mediated antinociceptive mechanism of propofol in the spinal cord. To investigate the interaction between propofol and spinal opioid receptors, Nadeson's group found that a δ opioid receptor antagonist, Naltrexone, inhibited the analgesic action of propofol at the lumbosacral level of the cord [81]. Feng et al. reported that propofol potentiated the depressant effect of alfentanil in isolated neonatal rat spinal cord and blocked Naloxone-precipitated hyper-responsiveness [82]. Additionally, spinal NMDA receptors were reported to be involved in the antinociceptive action of propofol. Xu et al. reported that intrathecal administration of an NMDA receptor agonist inhibited the antinociceptive effect of propofol; in contrast, an NMDA receptor antagonist enhanced the antinociceptive action of propofol [83]. These studies demonstrated that propofol has a synergistic action with several nociceptive transmission cascades including amino acid and opioid systems in the spinal cord.

### Potential molecular mechanisms of general anesthetics in inhibiting visceral pain

General anesthetics produce analgesia by acting on the spinal cord. But the precise mechanisms underlying visceral pain inhibiting by general anesthetics remain unclear. Recently, Kim et al. reported that neurons in the ventral spinal cord were more depressed by isoflurane, halothane, and propofol than neurons in the dorsal cord. It may suggest the possibility that PSDC neurons may be more vulnerably depressed by general anesthetics than neurons in superficial dorsal horn, since PSDC neurons are predominantly located in the vicinity of central canal in the spinal gray matter [84]. As summarized above, volatile anesthetics may act on spinal NMDA, AMPA, NK1 receptor, which are proved to be involved in the signaling transduction of PSDC neurons in visceral nociception. Therefore, we may suggest that volatile anesthetics possibly exert inhibitory effects on visceral pain transmission via those receptors in PSDC neurons. Similarly, commonly used intravenous anesthetic, propofol, may possibly exert the antinociception to visceral stimuli via the inhibition of NMDA receptors in PSDC neurons. GABA_A _and Glycine receptors are the important targeting sites of inhalational and intravenous anesthetics. However, whether GABA_A _and Glycine receptors are involved in signal transduction in PSDC neurons in visceral nociception remain to be determined. Additionally, it has been reported that isoflurane or propofol may influence various signaling transduction pathways such as PKC, CaMK, ERK, etc in hypoxic or normal cortex neurons [85-88]. However, few studies have been done to investigate the changes of signaling transduction pathways in spinal nociceptive neurons during general anesthesia. How general anesthetics affect the intracellular signaling transduction process in response to visceral pain in PSDC neurons remain to be investigated. Investigation of nociceptive signaling mechanisms of PSDC neurons involved in application of general anesthesia will advance our knowledge of clinical application of general anesthetics and help to identify new molecular targets for developing novel analgesic agents to manage visceral pain.

## Competing interests

The authors declare that they have no competing interests.

## Authors' contributions

YW participated in the design of the review and drafted the manuscript. JW, QL and HJN assisted with the preparation of the manuscript and figures. YY and LF conceived of the review, and participated in its design and helped to draft the manuscript. All authors read and approved the final manuscript.
